# Scaling Up the Production of Electrodeposited Nanowires: A Roadmap towards Applications

**DOI:** 10.3390/nano11071657

**Published:** 2021-06-24

**Authors:** Claudia Fernández-González, Jesús C. Guzmán-Mínguez, Alejandra Guedeja-Marrón, Eduardo García-Martín, Michael Foerster, Miguel Ángel Niño, Lucía Aballe, Adrián Quesada, Lucas Pérez, Sandra Ruiz-Gómez

**Affiliations:** 1 Departamento de Física de Materiales, Universidad Complutense de Madrid, 28040 Madrid, Spain; clafer03@ucm.es (C.F.-G.); aguedeja@ucm.es (A.G.-M.); 2 Instituto de Cerámica y Vidrio (CSIC), 28049 Madrid, Spain; jesus.guzman@icv.csic.es (J.C.G.-M.); a.quesada@icv.csic.es (A.Q.); 3 Instituto de Química Física Rocasolano—CSIC, 28006 Madrid, Spain; egarcia@iqfr.csic.es; 4 Alba Synchrotron Light Facility, 08290 Barcelona, Spain; mfoerster@cells.es (M.F.); mnino@cells.es (M.Á.N.); laballe@cells.es (L.A.); 5 Surface Science and Magnetism of Low Dimensional Systems, Universidad Complutense de Madrid, Unidad Asociada al IQFR-CSIC, 28040 Madrid, Spain

**Keywords:** nanowires, upscaling, anodization, electrodeposition, magnetic applications

## Abstract

The use of metallic nanowires is mostly reduced to scientific areas where a small quantity of nanostructures are needed. In order to broaden the applicability of these nanomaterials, it is necessary to establish novel synthesis protocols that provide a larger amount of nanowires than the conventional laboratory fabrication processes at a more competitive cost. In this work, we propose several modifications to the conventional electrochemical synthesis of nanowires in order to increase the production with considerably reduced production time and cost. To that end, we use a soft anodization procedure of recycled aluminum at room temperature to produce the alumina templates, followed by galvanostatic growth of CoFe nanowires. We studied their morphology, composition and magnetic configuration, and found that their properties are very similar to those obtained by conventional methods.

## 1. Introduction

Nanotechnology is one of the major driving forces behind the technological revolution of the 21st century, and nanomaterials play a key role in this revolution. In applications involving a large amount of nanomaterials, such as those related to nanomedicine [1,2], or for the fabrication of composites [3,4], nanoparticles—spherical nanomaterials—are commonly employed. These nanoparticles are normally synthesized by chemical methods [5] that are easily scaled-up, enabling real world applications [6]. Nanowires (NWs), which are much less widespread in applications than nanoparticles, have emerged in recent years, as nanomaterials have been called upon to play an important role in the development of breakthrough technologies in many fields, such as novel computation and data recording schemes [7,8], neuroscience [9,10], drug delivery [11,12], chemical sensing [13,14] or water splitting [15,16] among others. Electrodeposition is a very suitable growth technique for the fabrication of these nanomaterials. Using this technique, the properties of the NWs can be easily tailored [17,18] to adapt them to specific applications. In particular, taking advantage of their elongated shape, the magnetic properties of metallic NWs can be controlled just by tailoring their shape and composition [19,20]; this has been, for example, recently exploited in the fabrication of corrosion resistant composites [21], magnetoelectric nanocomposites [22] and bonded magnets [23]. In spite of having some promising performance, there are few NW-based magnetic applications on the market, due to the lack of a growth protocol allowing the fabrication of the large amounts of NWs needed to prepare a composite, preventing the transition from the laboratory proof of concept toward the industrial scale to take place.

The fabrication of electrodeposited NWs is normally based on the use of polycarbonate or anodized aluminium oxide (AAO) templates. The former are normally fabricated by ion-track etching of polycarbonate films irradiated with heavy ions [24]. Most of the research on the dispersion of NWs released from the template is based on the use of commercial templates, 6 μm thick, with a pore diameter between 30 and 100 nm. From a single template, less than 50 μg/cm${}^{2}$ of metallic NWs are obtained. In addition to the cost of the template, the main limitation comes from the low density of pores, which limits the quantity of NWs that can be grown, and the thickness of the templates, which limits the length of the NWs and, consequently, the mass of the grown material. Although very interesting for research purposes or dedicated applications, the expensive production is not suitable for mass production.

High-purity AAO templates are also widely used on the laboratory-scale production of NWs. In this case, the geometrical parameters of the templates can be fully controlled during the anodization process [25,26]. The control of the magnetic properties is normally linked to the production of well-ordered AAO templates with a diameter below 100 nm [27,28,29], which requires two-step anodization [30]. This procedure consists of two long electrochemical oxidation processes with a chemical etching step in between. After this process, a highly ordered structure of cylindrical NWs is formed. A low temperature is required during anodization (the solution must be between 0 and 5 ${}^{{}^{\circ}}$C) [31,32] and long anodization times are required (20–24 h for each anodization step) [32,33]. Therefore, although very versatile for research and lab-prototypes, the commercial application of NWs using AAO is still limited by their fabrication process, which is expensive, due to the high costs associated with the high-purity Al (99.999%) normally used as the starting material, as well as with the low temperature and large anodization time. Single-step anodization was studied by some authors as a modification of the most well-known two-step anodization with the main objective of reducing the synthesis time of alumina templates [34,35,36,37]. These approximations reduce the total anodization time, producing templates with pore arrangements that are far less ordered than those obtained using two-step anodization. Low purity aluminium was also explored as an alternative starting material for the synthesis of AAO templates [38,39,40,41]. In this case, low order was achieved, even after a two-anodization process, due to the low quality of the starting material.

Therefore, although partial approaches are found in the literature, there is not a comprehensive revision of the different approaches or a design of a full protocol, from the preparation of the templates to the growth of the NWs, allowing the mass production of NWs to fill in the gap between the lab developments and the real world applications. In this manuscript, we report a growth strategy to go one step closer toward large-scale applications of magnetic NWs by (1) increasing the quantity of produced NWs and (2) strongly reducing the costs and processing time. Focusing on the production of low-diameter ferromagnetic NWs for magnetic applications based on composites, we study the combined effect of using low purity Al templates and the one-step anodization process at room temperature. Moreover, the magnetic properties of the NWs were evaluated by PhotoEmission Electron Microscopy with X-ray Magnetic Circular Dichroism contrast (XMCD-PEEM), shown to be well suited for magnetic applications. Finally, to test the reported protocol, we fabricated a magnetic nanocomposite formed by NWs and nanoparticles with enhanced magnetic properties as a permanent magnet.

## 2. Materials and Methods

We used, as the starting material for anodization, three different type of Al foils: conventional AA1050 foils with 99.5%, recycled AA1050 foils, recovered from discarded waste from a mechanical workshop, and high-purity aluminum foils (99.999% Al, Goodfellow, Shanghai, China). Different steps were carried out to prepare the surface of recycled Al before the anodization process. First, the Al foils were deep cleaned, using acetone, ethanol and deionized water, followed by a polishing process, using a rotatory plate polisher (Struers DAP-V, Cleveland, OH, USA) working at 3000 rpm under water cooling with silicon carbide papers with different grit sizes (from #800 to #4000, Struers, Cleveland, OH, USA). After mechanical polishing, the sample was rinsed with deionized water to remove any possible sand reminders. The roughness of the surface was further improved by electrochemical polishing in a mixture of perchloric acid and ethanol (1:4 vol.) at a constant potential of 20 V for 5 min at 3 ${}^{{}^{\circ}}$C under constant stirring. In the case of the commercial aluminum, only electrochemical polishing was carried out before anodization.

Anodization was performed in a two-electrode electrochemical cell, at different temperatures, in a solution of 0.3 M oxalic acid (C${}_{2}$H${}_{2}$O${}_{4}$) under constant stirring. An adjustable DC power supply (EA Elektro-Automatik, Viersen, Germany) was used to control the applied voltage at a constant value of 40 V. As counter electrode, we used both a Pt mesh and carbon steel electrodes. After anodization, the remaining Al layer was removed by chemical etching, using a solution of 0.74 M CuCl${}_{2}$ and 3.25 M HCl, but leaving an Al frame in order to give mechanical support and to make the manipulation of the sample easier during the following steps. Finally, the pores were opened and the pore diameter was enlarged by etching under phosphoric acid solution (5% vol.) at room temperature for 2 h.

Electrodeposition was carried out in a three-electrode (with an Ag/AgCl electrode as reference) and two-electrode electrochemical cell controlled by an Ecochemie Autolab PGSTAT potentiostat, using a Pt mesh as a counter electrode. Before electrodeposition, a thin Au film was thermally evaporated on one side of the membrane to act as a working electrode. We used an electrolyte composed of CoSO${}_{4}$ (0.09 M), FeSO${}_{4}$ (0.1 M) and H${}_{3}$BO${}_{3}$ (0.4 M) as an additive. The pH was adjusted to 2.7, using H${}_{2}$SO${}_{4}$ 10% vol. After growth, the Au layer was removed, using a 0.1 M I${}_{2}$ and 0.6 M KI solution. Then, the alumina templates were dissolved, using a 0.4 M H${}_{3}$PO${}_{4}$ and 0.2 M H${}_{2}$CrO${}_{4}$ solution.

The nanoporous membranes were characterized by scanning electron microscopy (SEM). Images were taken with an accelerating voltage of 2 kV, using a ZEISS Sigma microscope (Zeiss, Jena, Germany). The average pore diameter, the interpore distance and the pore density were determined from SEM images with ImageJ, a freely available Java source code. The average thickness of the templates was determined from cross-section images.

Shadow XMCD-PEEM measurements were performed at the CIRCE beamline of the ALBA Synchrotron Light Facility [42,43,44]. Low-energy secondary electrons were used to form images of the spatially dependent X-ray Absorption Spectrum (XAS) in order to obtain chemical maps with lateral resolution of tens of nanometers. In addition, XMCD-images at the Fe and Co L${}_{3}$ absorption edges, obtained as the pixel by pixel subtraction of images acquired with opposite photon helicity, revealed the magnetization component along the X-ray incidence direction and thus the domain landscape of each NW. In principle, there are two different regions which show magnetic contrast: the wire itself and its shadow. Whereas the contrast in the wire reflects its surface magnetization, the magnetic contrast in the shadow comes from electrons photoemitted from the substrate, excited by the X-ray beam transmitted through the wire. It carries, thus, information of the magnetic configuration of the full NW width, i.e., mostly from the bulk.

## 3. Results and Discussion

### 3.1. Anodization of Recycled Aluminum

**Figure 1:**
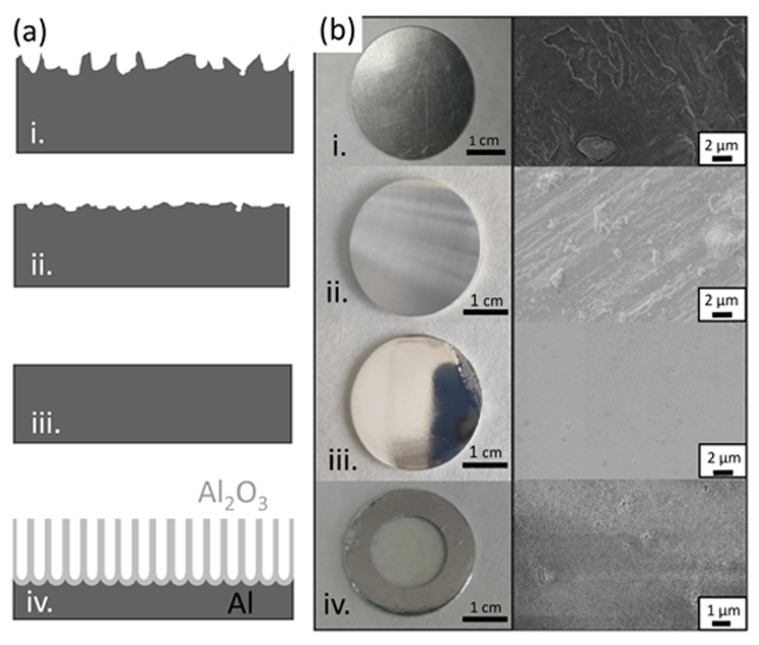
(**a**) Schematics of the cross-section of the aluminum samples during the alumina template preparation: (i) starting material, (ii) after mechanical polishing, (iii) after electrochemical polishing and (iv) after the anodization process and Al etching. (**b**) shows the surface morphology optical images (left) and scanning electron micrographs (right).

The first stage in the development of a protocol for the scaling up of the production of metallic NWs is the fabrication of AAO templates. It is important to take into consideration that our aim is developing a protocol to prepare small diameter NWs in solution, released from the AAO template, for applications in nanocomposites and, eventually, in biomedical applications. Therefore, the ordering of the nanopores in the AAO template is not relevant. One of our main strategies to reduce costs is the use of low-purity aluminum to produce AAO templates. To further reduce costs of the initial materials and increase sustainability by recycling waste material, we used as the starting material recycled, low-purity AA1050 aluminum from the waste of mechanical shops. We also used conventional AA1050 as well as high-purity aluminum for comparison purposes. Taking into account the origin of the starting material, different steps were carried out to prepare the surface before the anodization process, as described in the Materials and Methods section and summarized in Figure 1. After cleaning the surface, in the case of the recycled aluminum, large defects were clearly visible in both optical and SEM images (see step (i) in Figure 1). To avoid problems during anodization and to obtain alumina templates with homogeneous pore size distribution, a mechanical polish process was carried out. This process removed the macroscale defects, leaving a homogeneous Al surface, free of scratches and large surface defects (see step (ii) in Figure 1). After the mechanical polishing, the surface improved considerably, showing a better appearance with fewer scratches and defects in the macroscale (camera image) but was still rough in the microscale (SEM image). The roughness of the surface was further improved by electrochemical polishing (step (iii)). After this process, the surface showed a mirror appearance in both macro and microscale. Finally, the alumina template was fabricated through the anodization of Al (panel iv in Figure 1a), and the remaining Al layer was removed, leaving an Al frame for easier manipulation (see panel iv in Figure 1b).

Providing that the pore arrangement is not an important issue for the production of free standing NWs, we chose one-step anodization with the main objective of reducing the synthesis time for the preparation of the AAO templates. In the cited works [35,36,37], sulphuric and oxalic acids were used as electrolytes at temperatures from near 0 ${}^{{}^{\circ}}$C to 15 ${}^{{}^{\circ}}$C to obtain AAO templates with a pore diameter in the range of 20–50 nm. In this work, we explored the use of oxalic acid at room temperature (25 ${}^{{}^{\circ}}$C) because it is cost-effective in terms of time and investment—there is no need for cooling.

**Figure 2:**
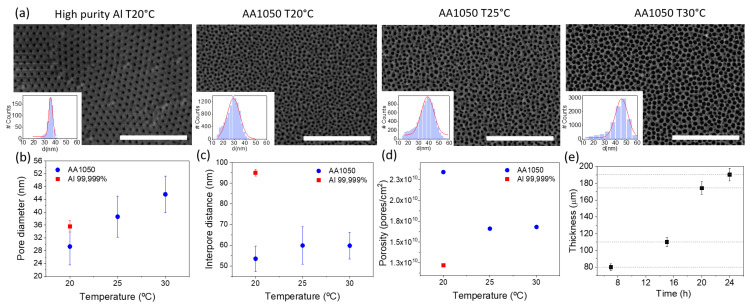
(**a**) Top-view images of AAO templates, anodized from different starting materials in 0.3 M oxalic acid, applying 40 V over 2 h at different temperatures. Histograms represent the pore size distribution and is calculated from different images, using ImageJ software. The scale bar is 1 μm in all images. (**b**) Pore diameter (**c**) interpore distance and (**d**) pore density vs. anodization temperature for Al 99.999 % and AA1050, calculated from the images in (**a**). Error bars are calculated from the standard deviation of the Gaussian fits of the histograms. (**e**) Evolution of the thickness of alumina templates as a function of the anodization time.

Different anodization experiments were conducted under the same electrochemical conditions in order to compare the effect of the Al purity as well as the selection of the composition of the counter electrode (Pt or carbon steel). In this latter case, no major differences were seen in the geometry and size of the nanopores when using Pt or steel as counter electrodes, as the final result was almost independent of the electrode. On the contrary, changing the starting material resulted in AAO templates with different structural parameters. Left panels of Figure 2a compare the top view of two AAO obtained from high-purity (99.999%) and low-purity (AA1050) Al. From the figure, it is clear that the pore density of AAO membranes obtained from high-purity aluminium is lower than those fabricated by anodization of low-purity Al. Therefore, when using AAO templates for electrodeposition, the use of low-purity aluminium allows producing a larger amount of NWs under the same conditions—due to the large pore density. However, although the mean pore diameter is very similar in both cases (see histogram), the shape of the pores is not as perfect as the one obtained with high-purity alumina resulting in a wider pore size distribution. The latter should thus be considered for applications in which a perfect shape of the NWs is needed.

We also studied the effect of temperature during the anodization process in order to evaluate the need for a well-controlled temperature environment. This is an important parameter considering the scaling up of the procedure, because the need for a temperature-controlled environment considerably increases the production costs. To that end, we carried out single-step anodization in 0.3 M oxalic acid, applying 40 V over 2 h, using recycled Al as the starting material at three different temperatures (20 ${}^{{}^{\circ}}$C, 25 ${}^{{}^{\circ}}$C and 30 ${}^{{}^{\circ}}$C). We show in Figure 2a top views of the different AAO templates together with the structural information extracted from the SEM images, as well as histograms calculated from different SEM images, using the ImageJ software. From SEM images, no evident morphological differences can be observed between the templates prepared at different temperatures.

Figure 2b shows the variation of the average pore diameter with the anodizing temperature. The pore diameter increases slightly with temperature. From the figure, an increase of less than 2 nm/${}^{{}^{\circ}}$C can be estimated. The average interpore distance for low-purity alumina remains more or less constant with the temperature, at lower values than those for high-purity alumina (Figure 2c), and the pore density decreases with the temperature (Figure 2d), a behavior that is consistent with the increase in the pore diameter observed before, after chemical etching. Since there is no significant change in the morphological parameters of the membranes, in all following experiments, anodization was carried out at room temperature without temperature stabilization.

Finally, the last parameter we studied is the anodization time, which is connected with the final thickness of the templates and, therefore, with pore length and the potential length of the NWs grown, using the membrane as template. Figure 2e shows the evolution of the pore length with the anodization time for templates made from low-purity Al. There is a clear linear relationship between the anodization time and thickness, reaching a thickness of 200 μm for 24 h of anodization time. The quality of the template is good for all anodization times, showing a dense array of parallel pores along the template. In spite of the linear behavior beyond 24 h, we limited the anodization times to this value since the use of membranes thicker than 200 μm may complicate the subsequent electrodeposition of the nanowires.

### 3.2. Growth of NWs for the Synthesis of Magnetic Nanocomposites

To show the potential of the upscaling procedure, we prepared ferromagnetic NWs, using the templates described above, to incorporate them into a nanocomposite. We describe the different steps for the upscaling of the fabrication of CoFe NWs with low diameters (∼50 nm) to ensure that they are magnetic single-domain nano-objects, which are needed for the particular application that we show as proof of concept (nanocomposites for bonded magnets). Similar NWs can be developed for different applications just by modifying the geometry of the AAO templates, the electrolytes and the growth conditions.

**Figure 3:**
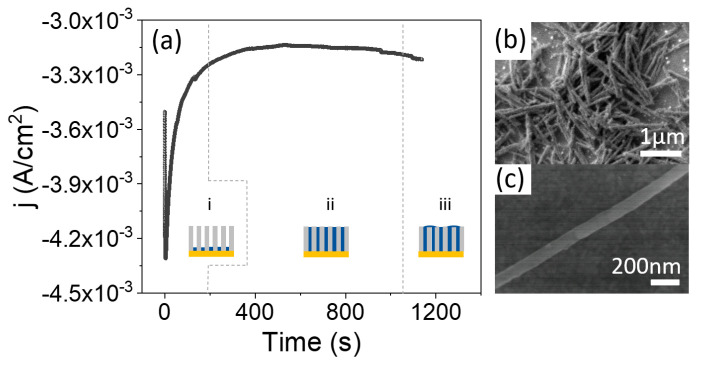
(**a**) Evolution of current density with time during the electrodeposition of CoFe NWs in a nanoporous alumina template. Three characteristic regions can be distinguished: (i) nucleation, (ii) NWs growth and (iii) overgrowth. (**b**,**c**) are SEM images of CoFe NWs at two different magnifications.

Galvanostatic electrodeposition, using only two electrodes, is far more convenient for scaling up the procedure and moving toward industrial applications. However, a calibration growth curve must be obtained in potentiostatic mode beforehand, using a three-electrode configuration, to determine the optimal current density for the growth of the nanowires. Figure 3a shows a calibration curve, obtained with an applied growth potential of −1.1 V, together with schematics of the evolution of the growth. We started with an AAO template prepared from recycled Al at room temperature. Au was thermally evaporated on one side of the template before electrodeposition. It can be seen in the calibration curve that, once nucleation of the CoFe in the bottom of the pores has taken place (region (i)), the current is stabilized to a constant value of −3.1 mA, corresponding to a current density of approximately 3 mA/cm${}^{2}$ (region (ii)), where the current density is calculated with respect to the total template area and not the active area. In this region (ii), the pores are uniformly filled with CoFe at a constant rate. In region (iii), the process was stopped before the current started to increase, reflecting that the pores were completely filled. The electrodeposition process should be stopped before reaching this region in order to avoid the formation of a CoFe thin layer over the NWs array.

Therefore, once the growth was calibrated, we grew Co${}_{35}$Fe${}_{65}$ NWs at room temperature under a constant current density of 3 mA/cm${}^{2}$. The growth time was calculated to completely fill the template. After growth, the Au layer was removed and the AAO templates were dissolved. As shown in Figure 3b,c, the nanostructures have a constant diameter along their full length. Finally, the NWs were transferred to ethanol for storage.

**Figure 4:**
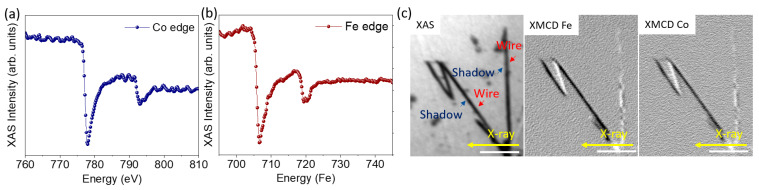
(**a**,**b**) X-ray absorption spectra of Co and Fe. (**c**) XAS image for CoFe NWs and XMCD image at Fe L${}_{3}$ and Co L${}_{3}$ edges for the same wires where it is possible to distinguish magnetic contrast only in the shadow of the wires. The scale bar is 1 μm.

One of the main problems of metallic NWs in applications is the potential oxidation during storage. In order to discard it, we studied the potential oxidation of NWs that had been stored in ethanol for three months. For this, a drop of the storage solution containing the NWs was deposited on a solid substrate (Si(111) with a thin Au buffer layer) and, after evaporating the solvent at room temperature, XAS spectra of Co and Fe were obtained from individual NWs by measuring stacks of PEEM images at different photon energies. Co and Fe average XAS spectra extracted from the middle of the shadow of the wires are shown in Figure 4a,b. Although the spectra are noisy, due to the small amount of material measured, the line shapes are clearly those of metallic Fe and Co with a main peak at the L${}_{3}$ edge and a wider peak at L${}_{2}$. There is only a slight hint of an oxidic component, visible as a small shoulder of the L${}_{2}$ peak, most likely coming from the partial oxidation of the wire surface.

To finish the characterization of the NWs, it is important to take into account that, to consider ferromagnetic NWs for the development of nanocomposites behaving as bonded magnets, the magnetization in the NWs should be axial, forming a single magnetic domain. To study the magnetic configuration of the NWs, we performed shadow XMCD-PEEM microscopy [45,46]. The left panel of Figure 4c shows the XAS image obtained at the Fe L${}_{3}$ edge with one photon polarization. Due to the small diameter of the NWs, in this case, only the shadow is clearly visible. The central and right panels show the XMCD images at the Fe L${}_{3}$ and Co L${}_{3}$ absorption edges, respectively. The white and black contrast in XMCD-PEEM images corresponds to areas with magnetization parallel or antiparallel to the incident X-ray beam direction, marked in the image with a yellow arrow. In this case, the direction of the incident photon beam is nearly perpendicular to the axis of one of the NWs and at 45${}^{{}^{\circ}}$ to the other ones. The wires at 45${}^{{}^{\circ}}$ display a single domain state with a strong contrast compared with the wire perpendicular to the beam. The latter has two distinct regions in the shadow along the width, with opposite contrast. Thus the magnetization in the NWs mostly lies along its long axis with a small vortex or curling component. In the wire perpendicular to the beam incidence, the small component of the magnetization that is not parallel to the wire long axis is probed, and can be seen to switch sense along the wire, forming magnetic domains with the same axial direction but with opposite chirality. Comparing the contrast of the differently oriented wires, we estimate that the deviation of the magnetization from the wire axis is around 18${}^{{}^{\circ}}$. This structure, consisting of the magnetization of almost the single domain along the NW axis, is very promising for magnetic applications. All above described experiments, carried out in individual NWs three months after fabrication, demonstrate that storage in ethanol ensures the conservation of the synthesized NWs, preventing them from oxidation and keeping their magnetic properties, suitable for application as a component of a magnetic nanocomposite.

**Figure 5:**
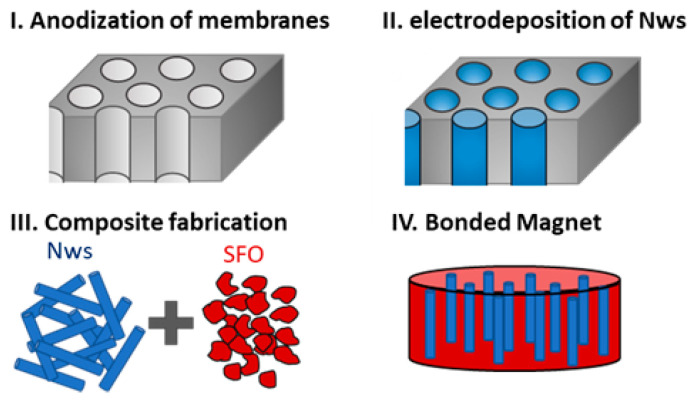
Schematic of the steps followed to fabricate the composite bonded magnet [23].

Once the NWs were fully characterized, we evaluated their potential use for the fabrication of composites containing NWs for applications as permanent magnets. In particular, we prepared composite bonded magnets made of CoFe NWs and strontium ferrite (SFO) particles (following the procedure described in Figure 5) in which the NWs are produced by electrodeposition, whereas the SFO nanoparticles are produced by chemical synthesis. Including magnetic NWs, these composites are interesting because NWs increase the saturation magnetization of the composite without decreasing the coercivity, which may result in an increase in the energy product (the figure of merit of permanent magnets). To compare the efficiency of the process in term of both cost and time, we grew electrodeposited nanowires, using different templates. In addition to the AAO templates, we also used commercial polycarbonate templates because, as mentioned in the introduction, these are one of the templates that are more commonly used in applications in which the nanowires are released from the template and used as individual nanomaterials.

Table 1 presents a comparison of the production of NWs, using the different materials and methods reported in this work for the preparation of the templates. It should be noted that we included under price only the costs related to the starting Al material and under time, that needed for anodization. Although polycarbonate templates are the best in terms of fabrication time (focusing only on the template synthesis because it is the time-limiting step of NW production), they are not suitable for industrial applications because the amount of NWs obtained is very low: we needed to use more than one hundred templates to prepare a nanocomposite using these templates.This is basically connected with the low pore density of 10${}^{8}$ cm${}^{-2}$ and low thickness of the polycarbonate template. The use of AAO templates increases the production of NWs by two orders of magnitude. Choosing the proper anodization process, it is possible to reduce the production time and, more importantly for industrial applications, to reduce the production costs by more than three orders of magnitude, just by the selection of the appropriate template. In spite of the larger dispersion in the diameter of the AAO templates using recycled Al as the starting material, the energy product of all magnetic nanocomposites is the same: 44% with respect to the counterpart bonded magnet made using only SFO nanoparticles [23,47]. Therefore, it is worth noting that the procedure described here allowed us to increase the production rate of CoFe NWs suitable for applications in magnetic nanocomposites, from μg/week to mg/week, while decreasing fabrication costs. A similar procedure can be used for example, for the fabrication of nanocomposites for different applications [21,22] as well as for certain biomedical applications [48] in which a large amount of individual magnetic NWs in solution are need, paving the way toward the implementation of lab-based results in real world applications.

## 4. Conclusions

To sum up, we developed an efficient route, in terms of time and costs, to produce large amounts of metallic NWs, based on a soft anodization process of recycled aluminum at room temperature. Although the use of recycled aluminum slightly increases the dispersion of the pore diameter with respect to more conventional starting materials, the magnetic properties of the grown materials are very similar to their counterparts that are grown using conventional AAO templates, showing optimal properties for magnet applications. In the process, there is no need for temperature stabilization, which further reduces the cost of potential industrial implementation. We also experimentally demonstrated that the NWs can be stored in ethanol for several months without changing their structural or magnetic properties. This technological procedure can be easily modified to produce NWs of different compositions and geometries, and integrated in industrial production, paving the way toward the use of electrodeposited metallic NWs in real-world applications.

## Figures and Tables

**Table 1:** Material, synthesis procedure, synthesis time and price for three different nanoporous templates and quantity of NWs produced.

Material	Process	Time	Price	NWs Quantity
		(h)	(€/cm${}^{2}$)	(mg /cm${}^{2}$)
Polycarbonate	—	—	2	0.01
High purity Al	two steps	48	6	1.2
Low purity Al	single step	24	0.007	1.2
Low purity recycled Al	single step	24	0–0.002	1.2

## Data Availability

The data presented in this study are available on request from the corresponding authors.

## Sample Availability

Small quantities of metallic nanowires are available from the authors.

## Abbreviations

The following abbreviations are used in this manuscript:

NW	nanowire
AAO	anodized aluminum oxide
SEM	scanning electron microscopy
XAS	X-ray absorption spectrum
XMCD-PEEM	PhotoEmission Electron Microscopy with X-ray Magnetic Circular Dichroism contrast
